# Network Topography Alterations in Alzheimer's Disease: Insights From Motif Changes via Multisite Datasets (*N* = 3262)

**DOI:** 10.1111/cns.70428

**Published:** 2025-06-19

**Authors:** Juntao Zhao, Yunyun Duan, Kun Zhao, Hongwei Li, Dawei Wang, Hongxiang Yao, Bo Zhou, Jie Lu, Pan Wang, Yan Chen, Xi Zhang, Ying Han, Yong Liu, Zhengluan Liao

**Affiliations:** ^1^ School of Artificial Intelligence Beijing University of Posts and Telecommunications Beijing China; ^2^ Queen Mary School Hainan Beijing University of Posts and Telecommunications Hainan China; ^3^ Department of Radiology Beijing Tiantan Hospital, Capital Medical University Beijing China; ^4^ Department of Radiology Qilu Hospital of Shandong University Jinan China; ^5^ Department of Epidemiology and Health Statistics School of Public Health, Shandong University Jinan China; ^6^ Institute of Brain and Brain‐Inspired Science, Shandong University Jinan China; ^7^ Department of Radiology The Second Medical Centre, National Clinical Research Centre for Geriatric Diseases, Chinese PLA General Hospital Beijing China; ^8^ Department of Neurology The Second Medical Centre, National Clinical Research Centre for Geriatric Diseases, Chinese PLA General Hospital Beijing China; ^9^ Department of Radiology Xuanwu Hospital of Capital Medical University Beijing China; ^10^ Department of Neurology Tianjin Huanhu Hospital Tianjin China; ^11^ Department of Psychiatry People's Hospital of Hangzhou Medical College, Zhejiang Provincial People's Hospital Hangzhou China; ^12^ Department of Neurology Xuanwu Hospital of Capital Medical University Beijing China; ^13^ National Clinical Research Center for Geriatric Disorders Beijing China; ^14^ Center of Alzheimer's Disease, Beijing Institute for Brain Disorders Beijing China; ^15^ Center for Inspur‐BUPT Beijing University of Posts and Telecommunications Beijing China

**Keywords:** Alzheimer's disease, brain network, motif, regional radiomics similarity network

## Abstract

**Background:**

Convergent studies have demonstrated that the topological structure of the brain network undergoes significant alterations in Alzheimer's Disease (AD). However, the underlying mechanisms driving these topological changes remain unclear. Network motifs, as fundamental components of brain networks, provide valuable insights into how disconnections may lead to alterations in the macroscale topological structure.

**Methods:**

This study focuses on undirected triangle motifs within the brain network, identifying 20 unique undirected triangle motifs based on edge strength and length derived from the regional radiomics similarity network (R2SN). To comprehensively capture the distribution of these motifs, we introduce a measure named Principal Motif Value (PMV) of the 20 motifs using principal component analysis (PCA).

**Results:**

Our findings reveal significant spatial heterogeneity in PMV across different brain regions. In addition, we identify reproducible alterations of PMV in AD, which were observed through three independent datasets, particularly in the inferior temporal gyrus, middle temporal gyrus, and parahippocampal gyrus. Notably, PMV demonstrates significant correlations with clinical manifestations and neurological features. Finally, we elucidate that alterations in PMV are associated with gene expression related to neuronal systems and synapsis features.

**Conclusion:**

These findings provide novel insights into the relationship between disconnection and the topological structure of the brain network in AD.

## Graphical abstract

This study identifies 20 undirected triangle motifs in the brain network using edge strength and length from the regional radiomics similarity network (R2SN) and introduces the Principal Motif Value (PMV) via principal component analysis (PCA). PMV significantly correlates with clinical and neurological features and is linked to gene expression in neuronal and synaptic systems, offering novel insights into brain network topology and disconnection in Alzheimer's disease (AD).

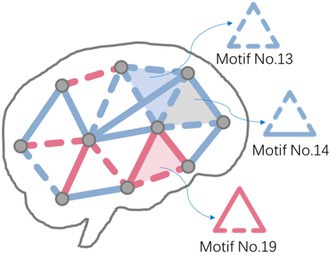

## 1 Introduction

Alzheimer's Disease (AD) is one of the most common neurodegenerative disorders in older adults. Fundamental theories in neuroscience have demonstrated that the dysfunction of the brain network has served as a hallmark of AD [1]. Understanding the biological mechanisms linking altered brain structure to cognitive decline is crucial for developing effective therapeutic strategies for AD. According to the classical connectome framework, billions of interconnected neurons in the human brain form an intricate and dynamic network [2], enabling efficient information processing and integration. This perspective provides a critical model for understanding the complex nature of cognitive functions [3, 4]. The motif, as a fundamental structural unit in networks [5], is analogous to a cell in the human body, offering a robust framework grounded in graph theory [6, 7], and thus serves as a crucial tool for investigating structural alterations in brain networks. Building upon these insights, leveraging motifs to investigate microstructural changes within brain networks is essential for understanding how these networks influence cognition.

Network neuroscience has traditionally emphasized node‐centric analyses, focusing on regional specialization [8] while largely treating edges as secondary features. In contrast, the role of edges has received comparatively less attention. Although edge properties provide essential information for evaluating nodal network characteristics, they are rarely considered fundamental organizational descriptors of brain networks. In contrast, motifs are subgraphs with a fixed number of nodes, characterized by distinct edge patterns. The distribution of motifs reveals the local building blocks of the network, offering insights into its underlying structure [9, 10]. By assessing motif pattern frequencies using surrogate networks, the under‐ and over‐expression of specific motifs can be quantified [11, 12] and can also be correlated with the main dimensions of network organization [13], thereby uncovering the structural foundations that support network functionality. Motifs provide a robust framework for reducing network complexity, focusing on smaller, recurrent patterns that break down large‐scale networks into more manageable and analyzable components. This method enables a deeper understanding of how localized interaction patterns at the micro level influence the emergent structural and functional properties of networks at the macro scale. When applied to the study of AD, this framework enables a more refined exploration of the biological mechanisms driving connectivity loss, facilitating insights into how micro‐level changes contribute to the overall network disruptions observed in AD.

Traditional brain networks are constructed from various sources, such as fMRI [14] and DTI [15, 16]. However, these networks often suffer from limited signal‐to‐noise ratios and insufficient robustness, which significantly compromises the reproducibility of research findings. Although considerable progress has been made in developing diverse brain network construction methods, there remains a critical need for a well‐validated, widely accessible model that can accurately map individual‐level anatomical brain network architecture. In recent years, novel sMRI‐based morphological networks have increasingly attracted the attention of researchers, with the Regional Radiomics Similarity Network (R2SN) playing an irreplaceable role. In contrast to using cortical thickness [17] or gray matter volume [18] as features, R2SN, on account of radiomics similarity, provides a quantitative portrayal of properties specific to the interior of brain regions [19]. R2SN has been successfully applied to characterize multiple psychiatric disorders through abnormal brain patterns [20, 21, 22]. Thus, it is possible to discover the rules concealed beneath motif distributions based on the R2SN matrix.

In this study, we aimed to elucidate macro‐level topological alterations in AD brain networks through the lens of motifs. Utilizing the R2SN network, we defined a novel topological structure‐based biomarker, Principal Motif Value (PMV), which was employed to characterize micro‐structural distributions and transitions within the brain. We further validated the clinical relevance of this biomarker by assessing its correlations with key clinical indicators. Additionally, we explored neurobiological associations by investigating gene enrichment, high‐risk gene correlations, and spatial relationships with various PET/SPET receptor maps. Collectively, our findings establish PMV as a robust and consistent topological biomarker, shedding light on how microstructural changes influence macro‐scale brain networks, thereby offering novel insights into the pathophysiology of AD and other neurodegenerative disorders.

## 2 Materials and Methods

### 2.1 Participants

The primary discovery dataset utilized in the present study is derived from the Alzheimer's Disease Neuroimaging Initiative dataset (ADNI), comprising 1654 participants, including 605 normal controls (NC), 766 individuals with mild cognitive impairment (MCI), and 283 patients with AD. Detailed demographic and clinical characteristics of the participants are provided in Table 1.

**TABLE 1:** Demographic and clinical characteristics of participants in the ADNI dataset.

Group	Age (years) ± SD	Sex (M/F)	Clinical measure ± SD
MMSE (*n* = 1654)			
NC (*n* = 605)	73.47 ± 6.16	279/326	29.08 ± 1.10
MCI (*n* = 766)	72.96 ± 7.69	450/316	27.57 ± 1.81
AD (*n* = 283)	74.91 ± 7.70	152/131	23.18 ± 2.14
*p*	< 0.001	< 0.001	< 0.001
Adas‐cog11 (*n* = 1652)			
NC (*n* = 605)	73.47 ± 6.16	279/326	6.97 ± 3.07
MCI (*n* = 765)	72.98 ± 7.68	449/316	10.41 ± 4.42
AD (*n* = 282)	74.88 ± 7.70	151/131	19.65 ± 6.66
*p*	< 0.001	< 0.001	< 0.001
Adas‐cog13 (*n* = 1643)			
NC (*n* = 603)	73.49 ± 6.16	279/324	10.36 ± 4.38
MCI (*n* = 762)	72.97 ± 7.69	448/314	16.64 ± 6.66
AD (*n* = 278)	74.93 ± 7.66	148/130	30.03 ± 7.91
*p*	< 0.001	< 0.001	< 0.001
Adas‐Q4 (*n* = 1654)			
NC (*n* = 605)	73.47 ± 6.16	279/326	2.74 ± 1.76
MCI (*n* = 766)	72.96 ± 7.69	450/316	5.41 ± 2.53
AD (*n* = 283)	74.91 ± 7.70	152/131	8.64 ± 1.49
*p*	< 0.001	< 0.001	< 0.001
Avlt‐1 (*n* = 1650)			
NC (*n* = 603)	73.46 ± 6.17	278/325	45.34 ± 9.95
MCI (*n* = 766)	72.96 ± 7.69	450/316	34.52 ± 10.76
AD (*n* = 281)	74.85 ± 7.69	150/131	23.01 ± 7.66
*p*	< 0.001	< 0.001	< 0.001
Avlt‐2 (*n* = 1650)			
NC (*n* = 603)	73.46 ± 6.17	278/325	6.03 ± 2.40
MCI (*n* = 766)	72.96 ± 7.69	450/316	4.07 ± 2.59
AD (*n* = 281)	74.85 ± 7.69	150/131	1.80 ± 1.76
*p*	< 0.001	< 0.001	< 0.001

To ensure the robustness of our study's findings, we utilized two independent validation datasets: the European DTI Study on Dementia (EDSD) and the in‐house Multi‐Center Alzheimer Disease Imaging Consortium dataset (MCADI). The EDSD dataset comprises 573 participants, including 230 NC individuals, 183 with MCI, and 160 diagnosed with AD. Similarly, the MCADI dataset includes 1035 participants, with 336 NC, 300 MCI, and 399 AD subjects. A comprehensive summary of the demographics of the participants is presented in Table 2. This study received ethical approval from the Biomedical Ethical Review Committee of Beijing University of Posts and Telecommunications (Approval No. 202402016).

**TABLE 2:** The summary of participants in this study.

Dataset	Group	Age (year) ± SD	Sex (M/F)
ADNI (*n* = 1654)	NC	73.47 ± 6.16	279/326
	MCI	72.96 ± 7.69	450/316
	AD	74.91 ± 7.70	152/131
EDSD (*n* = 573)	NC	68.76 ± 6.14	108/122
	MCI	71.46 ± 7.02	83/100
	AD	72.52 ± 8.05	68/92
MCADI (*n* = 1035)	NC	64.71 ± 8.86	148/188
	MCI	68.49 ± 9.28	138/162
	AD	69.23 ± 9.16	154/245

### 2.2 Data Preprocessing and Principal Motif Value Computing

**FIGURE 1:**
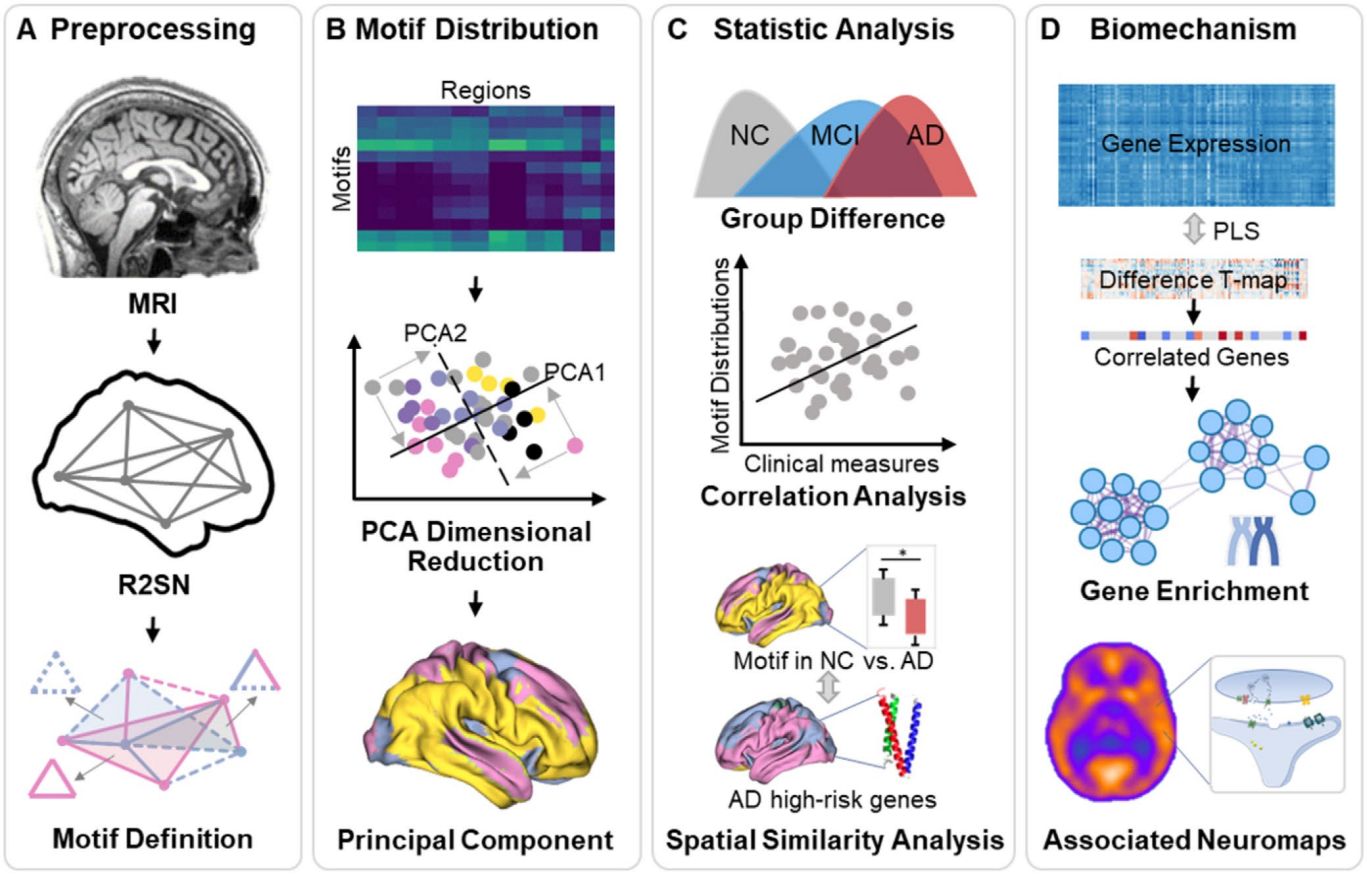
The workflow of the research. (A) (B) First, structural magnetic resonance imaging (sMRI) data of 1654 participants from the Alzheimer's Disease Neuroimaging Initiative (ADNI) were analyzed using R2SN‐based morphometry to calculate the Principal Motif Values (PMV) as biomarkers. (C) Subsequent exploratory analyses, including group comparisons, correlation analyses with clinical measures, and spatial similarity assessments, were conducted to demonstrate the robust association of PMV with Alzheimer's Disease (AD). (D) Second, we explored the underlying biomechanisms by performing gene enrichment analysis and PET/SPET receptor maps correlations to elucidate the connection between PMV and biological pathways involved in AD pathogenesis. Finally, replication studies were conducted on the EDSD and MCADI datasets to assess the reproducibility and consistency of the findings, further validating the reliability of PMV as a biomarker for AD.

Structural MRI scans with T1‐weighted sequences underwent N4 bias field correction and denoising. The images were then spatially normalized to the Montreal Neurological Institute (MNI) template using the “SyN” algorithm implemented in Advanced Normalization Tools (ANTs). Then we extracted a comprehensive set of radiomic features for each brain region, as defined by the Brainnetome atlas [23], to construct a regional morphology profile. This included 14 intensity‐based features and 33 texture‐based features [24]. To reduce redundancy and mitigate multicollinearity, we applied correlation‐based feature selection, retaining 25 radiomic features for subsequent analysis. Following min‐max normalization, individual Regional Radiomics Similarity Networks (R2SN) were constructed by computing pairwise Pearson correlation coefficients between regional radiomic profiles. This novel framework provides enhanced robustness through radiomic feature selection, biological plausibility through anatomical constraint, and a new perspective for understanding brain organization at the individual level. In this study, motif profiles, consisting of three nodes and their corresponding interlinked edges, were used to represent the topography of the R2SN. After removing duplicate motif types, including those that are identical when rotated or flipped, these motifs were further classified into 20 types based on connection length and connection strength (Figure S1). Both connectivity measures were binarized based on their median values across the entire cohort: connection length (Euclidean distance in MNI space, median = 76.11) and connection strength (R2SN edge weight, median = 0.75, SD = 0.05), as shown in Figure 1. Participant‐level variation was preserved in this dichotomization. Through systematic exploration of the whole‐brain network, we identified and quantified all motif triangles. In this analysis, each of the three constituent brain regions contributed to the count for their respective motif type, ultimately yielding a matrix with dimensions of motif type × brain regions (Figure S2). To comprehensively capture the distribution of these motifs, we applied principal component analysis (PCA) and computed the first principal component, termed the Principal Motif Value (PMV), to encapsulate the distribution of all 20 motifs across the brain rather than a single motif. To quantify cross‐motif covariation patterns across brain regions, we derived the Principal Motif Value (PMV) using the following PCA‐based procedure.

#### 2.2.1 Data Standardization

Given a motif count matrix $X\in {\mathbb{R}}^{20\times 246}$(where rows represent 20 motifs and columns represent 246 brain regions), we standardize each feature (brain region) to zero mean and unit variance:

$$Z_{\mathrm{ij}}=\frac{X_{\mathrm{ij}}-\mu_{j}}{\sigma_{j}},i=1,\ldots ,20;j=1,\ldots ,246$$

where $\mu_{j}$ and $\sigma_{j}$ denote the mean and standard deviation of motif counts for the j‐th brain region.

#### 2.2.2 Covariance Matrix Computation

The covariance matrix $C\in {\mathbb{R}}^{246\times 246}$ quantifies linear dependencies between brain regions:

$$C=\frac{1}{n-1}Z^{T}Z$$

Here, n=20 (number of motifs) and Z is the standardized matrix.

#### 2.2.3 Eigenvalue Decomposition

We decompose C into its principal components:

$$C=V\Lambda V^{T}$$

where V is the orthogonal matrix of eigenvectors (columns = principal directions), Λ is the diagonal matrix of eigenvalues (sorted in descending order), reflecting explained variance.

#### 2.2.4 Dimensionality Reduction

Retain the first principal eigenvector $v_{1}\in {\mathbb{R}}^{246}$ (associated with the largest eigenvalue $\lambda_{1}$) to capture maximal variance. The Principal Motif Value (PMV) is computed by projecting Z onto $v_{1}$:

$$\mathrm{PMV}=Zv_{1}$$

The resulting $\mathrm{PMV}\in {\mathbb{R}}^{246}$ represents a univariate summary of cross‐motif variance for each brain region.

### 2.3 The Altered Pattern of the PMV in the MCI and AD Groups

To evaluate PMV alterations in the MCI and AD groups, we first assessed the normality of the data using Shapiro–Wilk tests, which indicated significant deviations from normality in all three groups (NC, MCI, and AD; *p* < 0.05). Given the non‐normal distribution, we employed the Kruskal‐Wallis test, a nonparametric analog of one‐way ANOVA, to compare PMV across the three groups. Upon identifying significant group differences (*p* < 0.05, Bonferroni correction), we conducted post hoc Mann–Whitney tests with Bonferroni correction for pairwise comparisons between significant regions. While the U statistic quantifies rank‐order differences, its standardized variant—the *Z*‐score—was derived to facilitate interpretation and effect size estimation, following established normalization approaches. The *Z*‐score was computed as:

$$Z=\frac{U-\mu_{U}}{\sigma_{U}}$$

where U is Observed Mann–Whitney U statistic, $\mu_{U}=\frac{n_{1}n_{2}}{2}$ expected mean under the null hypothesis of identical distributions, $\sigma_{U}=\sqrt{\frac{n_{1}n_{2}\left(n_{1}+n_{2}+1\right)}{12}}$ standard deviation of U, accounting for sample sizes $n_{1}$ and $n_{2}$.

Those altered patterns were verified based on two independent datasets. To further explore the biological basis of these alterations, we computed Pearson correlations between PMV and clinical measurements, including CSF biomarker (Aβ, Tau, P‐tau), FDG, cognitive ability (ADAS‐cog11, ADAS‐cog13, AVLT1, AVLT2, and MMSE scores) in the MCI and AD groups.

### 2.4 The Gene Enrichment Analysis for the Altered PMV

To explore the potential genetic basis, we performed a Partial Least Squares (PLS) correlation analysis between the *Z*‐map of altered PMV in AD and gene expression data. Whole‐brain gene expression data were obtained from the Allen Human Brain Atlas (AHBA) and aligned to the Brainnetome Atlas via the “Abagen” toolbox [25, 26, 27]. Since the AHBA dataset includes only two right hemisphere samples, we utilized data from the left hemisphere exclusively and used “centroids” to impute missing regions. The correlation statistical significance was determined after performing permutation and spin tests for correction. Furthermore, the top 500 genes, ranked by absolute *Z*‐scores, were selected for subsequent gene enrichment analysis to explore associated biological processes via Metascape.

### 2.5 The Relationship Between Altered PMV and AD‐Related High‐Risk Genes and Specific Receptors

Besides, we also computed the relationship between the *Z*‐map of altered PMV in AD and AD‐related high‐risk genes presented on the AHBA website, a set that includes A2M, ACE, ACHE, APBA1, APBB2, APLP1, APLP2, APOC1, APP, BACE2, BCHE, BLMH, CASP3, CHRNA3, CTSB, DBN1, ESR1, GSK3B, IL1B, KCNIP3, KLK6, LRP1, LRRC15, MAPT, PLAU, PSEN1, PSEN2, and SORL1. Additionally, to further determine which synaptic systems were related to the PMV, we utilized the JuSpace toolbox [28], a software package designed to integrate different imaging modalities with positron emission tomography (PET)‐derived neurophysiological measures to calculate the correlation between the PMV and the receptor maps.

## 3 Results

### 3.1 Distribution Differences With Brain Functional Systems and Anatomic Regions

**FIGURE 2:**
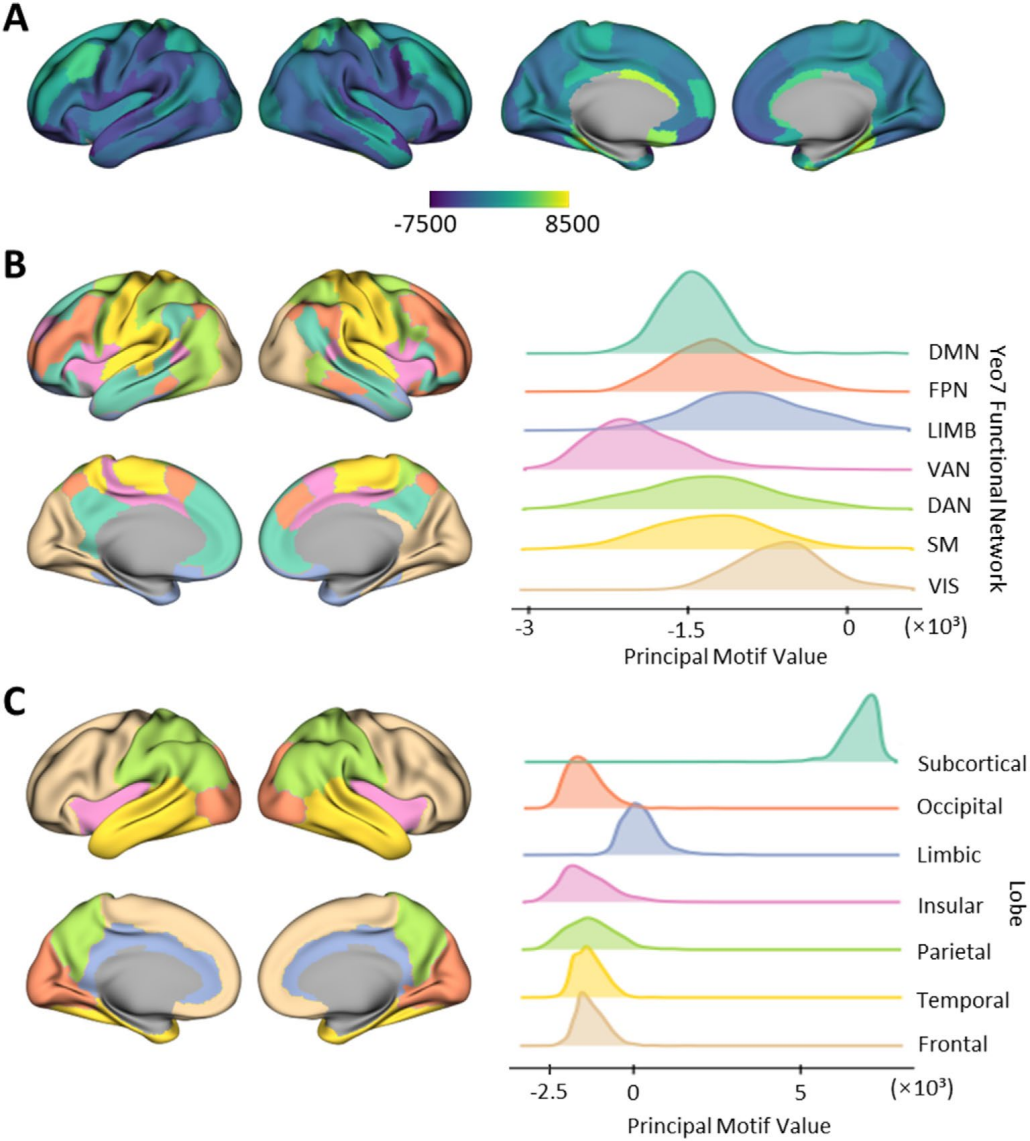
The PMV distribution on the cerebral cortex. (A) The average PMV for the NC group in the ADNI dataset. (B and C) PMV distribution for the NC group in the ADNI dataset, presented in functional systems and anatomical regions. Left: An illustration of the distribution map. Right: The probability distribution of PMV. DAN, dorsal attention network; DMN, default mode network; FPN, frontoparietal network; LIMB, limbic network; SM, somatomotor network; VAN, ventral attention network; VIS, visual network.

**FIGURE 3:**
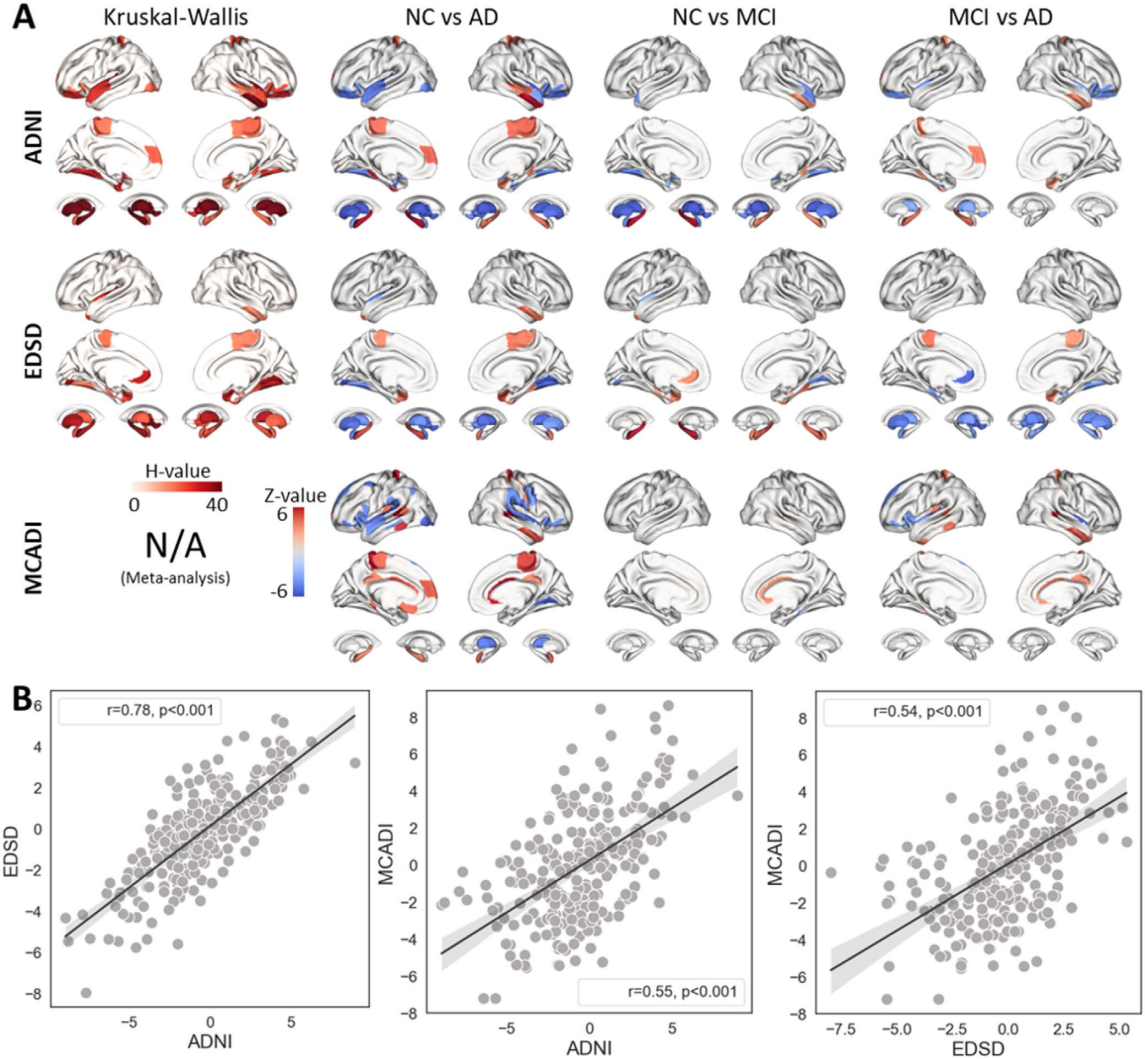
The difference map of the PMV across groups and datasets. (A) Three rows represent different datasets, including ADNI, EDSD, and MCADI. The first column shows the H‐value of the Kruskal‐Wallis test (*p* < 0.05, Bonferroni correction; MCADI was not applicable due to the application of meta‐analysis across multiple centers). The remaining columns demonstrate the difference maps across groups. (B) *Z*‐value correlations of the differences between the AD and NC groups across datasets.

Using the sMRI data as described previously, each participant was characterized by a PMV, represented as a 246‐element vector corresponding to motifs across 246 brain regions. The PMV demonstrated a regional clustering distribution throughout the brain (Figure 2A). The distribution of PMV in the normal control (NC) group is shown in Figure 2A. Notably, as illustrated in Figure 2C, the subcortical regions exhibited significantly more positive PMV differences compared to cortical areas, while the limbic system showed higher PMV values among cortical regions (Figure 2B). We also calculated the distribution of PMV across functional systems defined by the Yeo networks. Regions within the limbic lobe displayed slightly elevated PMV values relative to other lobes. In the Yeo 7 network [29], distinct PMV distributions were observed across functional networks, with the visual network (VIS) exhibiting a higher trend relative to the average, while the ventral attention network (VAN) demonstrated lower values (Figure 3C).

### 3.2 Replicability and Consistency Altered Patterns of PMV in the AD and MCI Groups

The Kruskal‐Wallis test results indicate that the inferior temporal gyrus, middle temporal gyrus, parahippocampal gyrus, and fusiform gyrus are primary regions with significant differences (*p* < 0.05/246). In the subcortical regions, nearly all areas exhibit substantial differences across groups (*p* < 0.05/246). Subsequent Mann–Whitney tests with Bonferroni correction revealed the direction of these changes. Compared to the NC group, the AD group exhibits positive changes in the inferior temporal gyrus, posterior cingulate cortex, and hippocampus. Conversely, the connections in the lateral temporal lobe, occipital lobe, parahippocampal gyrus, and thalamus show a negative trend as AD progresses. The comparison between the NC and MCI groups follows a similar pattern, although the statistical significance is weaker than that observed between the NC and AD groups (Figure 3A). Furthermore, those significant regions yielded from the ADNI dataset can be replicated in EDSD and MCADI datasets (Figure 3B). The correlation analysis of *Z*‐maps across the datasets further supports the robustness of the PMV patterns, with Pearson correlation coefficients of 0.78, 0.55, and 0.54, all with *p* < 0.001.

### 3.3 Notable Correlations Discrepancy Across Clinical Measurements

**FIGURE 4:**
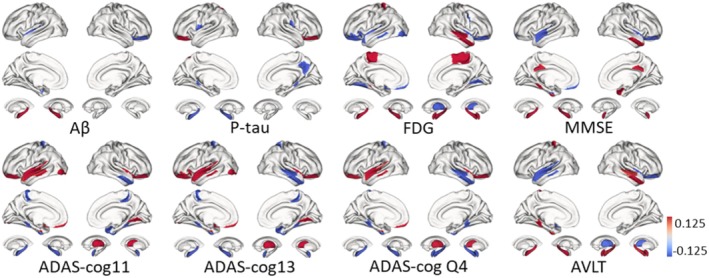
Significant regions of clinical measurements, including clinical scales and biological indicators, were identified. Correlations were calculated between diseased and non‐diseased individuals in the ADNI dataset, with *p* < 0.05, Bonferroni corrected.

We conducted Pearson correlation analyses between PMV and various clinical measurements, revealing that different clinical scores consistently highlight similar brain regions and trends (Figure 4). Nearly all clinical scores show a positive correlation with disease progression in the temporal lobe, frontal–parietal regions, and hippocampus, which aligns closely with the patterns observed in the PMV results from the Mann–Whitney tests. Negative correlations, typically indicated by blue in the visualizations, are predominantly observed in the occipital lobe and thalamic regions.

### 3.4 Underlying Association Behind Biological Mechanisms

**FIGURE 5:**
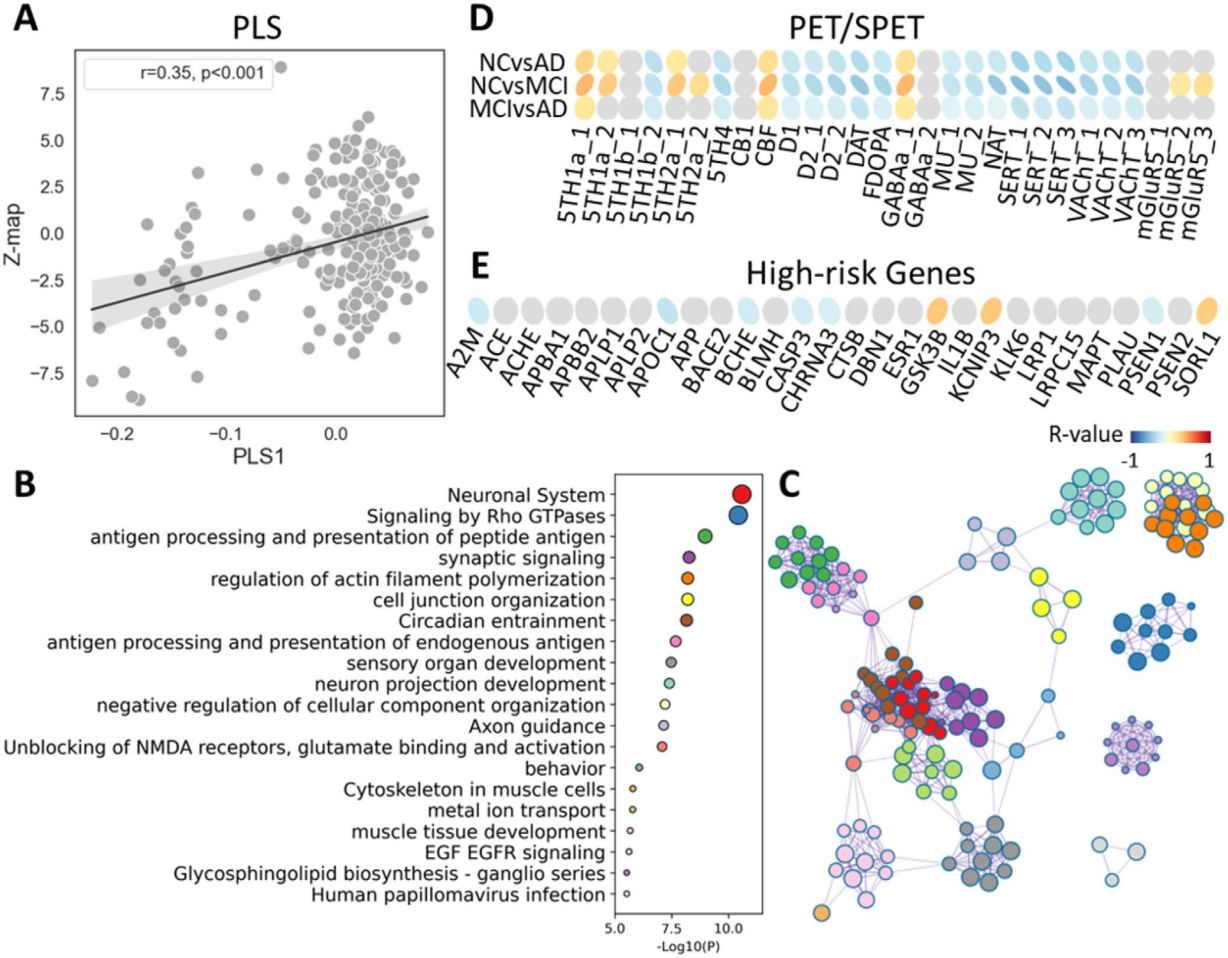
Gene enrichment analysis and correlations with PET/SPET receptor maps. (A) Correlation between the *Z*‐map of the PMV and PLS1 of the gene list obtained from the AHBA, *r* = 0.35, *p* < 0.001. (B and C) The top 20 significant GO terms calculated by Metascape and their connections with the top 500 genes, ranked by the absolute *Z*‐scores of their contributions to the first component. (D) Correlations between the *Z*‐map and spatial neurotransmitter maps; PET/SPET atlas data were obtained from the JuSpace toolbox. (E) Correlations between the Z‐map and high‐risk genes identified from the AHBA website.

The PLS result revealed that the first principal component was significantly correlated with PMV (*r* = 0.35, *p* < 0.001). This component accounted for 12.2% of the variance (p_perm_ = 0.011; Figure 5A). The enrichment results indicated that 20 significant Gene Ontology (GO) biological processes are closely related to the PMV (Figure 5B). The gene analysis emphasized the neuronal system, signaling as well as antigen processing and presentation, comprising the neuronal system, signaling by Rho GTPases, synaptic signaling, and antigen processing and presentation of peptide antigen (Figure 5C). To further investigate the molecular mechanism of PMV, we applied the PET/SPET receptor maps correlation analysis (Figure 5D). Compared to other receptors, the SERT and the DAT showed the most significant correlations between receptor maps and PMV. It is worth noting that the differences between NC and MCI are larger than those between NC and AD in almost all receptors. It is not, as our common sense would have us believe, that the difference gets more significant as the disease progresses. Moreover, among 28 high‐risk AD genes identified from the AHBA database, GSK3B, KCNIP3, and SORL1 were found to be positively correlated with PMV. Conversely, A2M, AOPC1, BCHE, CASP3, CHRNA3, and PSEN1showed a negative correlation with PMV (Figure 5E).

## 4 Discussion

Our study introduces the PMV as a novel neurological biomarker for elucidating the relationship between topological structure and connectivity loss in Alzheimer's Disease (AD). We have experimentally validated PMV's capacity to detect AD‐related topological alterations, providing a new framework for understanding the biological significance of structural transitions. These findings contribute to a deeper comprehension of the characteristic patterns of connectivity loss in AD and offer key insights into the underlying mechanisms driving these pathological changes.

Previous research has extensively investigated AD, with alterations in brain topological structure being recognized as a hallmark feature of the disease. However, the biological underpinnings of these topological changes remain poorly understood. Recent findings highlight the utility of graph theory as a robust approach for probing brain topology. Consistent with this perspective, our study systematically examined motif distributions in the brain, offering further evidence that microstructural patterns can reliably reflect macroscopic shifts in brain topology.

In this study, we applied Partial Least Squares (PLS) analysis to summarize the distribution of 20 motifs using a single value, with the first principal component accounting for an average of 65.34% of the variance across these motifs. Examination of component contributions revealed that the first principal component was primarily influenced by motifs 19, 13, and 14, accounting for 43%, 14%, and 12% of the PCA variance, respectively. These motifs exhibit similar distribution trends with both connection length and strength. Specifically, motif 19 is characterized by three strong and predominantly long connections, commonly observed in the cerebral cortex. In contrast, motif 13 and motif 14 consist of weak connections and a high proportion of short connections, frequently found in subcortical areas (Figure S3). As a result, the PMV indicated a greater concentration of short and weak connections, which means a more positive PMV value corresponds to a brain region characterized by weaker and shorter connections.

The observed differences between the cortex and subcortex are reflected in the anatomical mapping, where the subcortex exhibits pronounced deviations compared to cortical regions (Figure S4). Meanwhile, regions within the limbic lobe, which serves as a transitional interface between the cerebral cortex and subcortex, display slightly elevated PMV values compared to other cortical regions [30]. These disparities are primarily attributable to the inherent structural variations between the cortex and subcortex [31]. Specifically, synaptic distribution in the cortex is characterized by a high degree of hierarchical organization and regionalization, facilitating the formation of intricate local neural circuits [32]. In contrast, synaptic connections in the subcortex are more specialized, with less pronounced hierarchical differentiation [33]. Cortical synapses predominantly occur between distinct cortical layers and are confined to intra‐cortical interactions, whereas subcortical synapses primarily involve connections between the cortex and subcortical nuclei, or within subcortical regions themselves.

In the analysis of brain functional systems, the Yeo 7 network, which encompasses exclusively cortical regions [29], consistently shows negative PMV values across all seven networks. This pattern suggests that highly interconnected brain regions, particularly those responsible for sensory processing and motor control, require more efficient local connectivity to facilitate rapid neural processing. A notable example is the default mode network (DMN), which plays a crucial role in integrating information from disparate brain regions to support complex cognitive activities, such as introspection and memory retrieval. The relatively weaker connectivity observed in the DMN may reflect its reliance on long‐range connections for the integration of information across diverse neural circuits, rather than on densely clustered local connections [34]. This pattern aligns with the small‐world topology of the brain, where strong local connections facilitate efficient processing, while weaker global connections support information integration and large‐scale neural coordination [35]. Such a structural organization maximizes the efficiency of information transfer across the brain, optimizing performance across a wide range of cognitive tasks [36]. The structural and functional differences between cortical and subcortical regions, as well as the specialized connectivity within networks, underscore the utility of PMV as a robust biomarker for elucidating the neural mechanisms underlying a wide range of cognitive activities.

Global properties of network organization, such as increased randomness in network organization or lower clustering coefficients, have been shown to reflect brain atrophy in AD [37, 38, 39]. The changes in PMV, as previously discussed, are directly related to these graph‐theoretical properties, providing an opportunity to compare our findings with those from other studies. In the AD group, compared to the NC group, positive PMV changes were observed in the inferior temporal gyrus, posterior cingulate cortex, and hippocampus. These changes suggest that connections in these regions become shorter and weaker, with reduced integration with other areas. Conversely, connections in the lateral temporal lobe, occipital lobe, parahippocampal gyrus, and thalamus exhibit strengthening as AD progresses, particularly in the medial temporal lobe, which is associated with cognitive impairments [40]. Comparisons between the NC and MCI groups reveal a similar trend, though the changes are less pronounced than in the AD group, which aligns with the disease's progression. Specifically, increased activity in the precuneus correlates with accelerated memory decline, while similar patterns in the temporal and prefrontal lobes are also linked to memory deterioration [41]. Additionally, the observed blue color in the temporal gyrus and temporal pole in the MCI group, which indicated stronger connection, aligns with findings from other studies compared to NC [42].

Across three different datasets, the PMV demonstrates consistent transitions and variation trends among various groups, even when considering results from meta‐analyses. All three datasets exhibit strengthened connections in the lateral temporal cortex and medial temporal lobe, along with a weakening trend in the parietal lobe and hippocampus. Additionally, even at the early stage of MCI, the PMV reveals shifts in topological structure, particularly in the hippocampus and temporal lobe, which are well‐established as key regions in AD pathology. These results demonstrate that the PMV changes associated with AD progression are consistent with previous research, highlighting the PMV's sensitivity for detecting early structural changes, further supporting PMV as a reliable biomarker for tracking disease‐related alterations.

Our gene enrichment analysis further supports the biological relevance of PMV; the results indicated that PMV is strongly associated with the neuronal system and signaling bioprocesses, both of which are critically implicated in the pathophysiology of AD. Additionally, correlation analyses were also conducted on high‐risk genes implicated in AD found in AHBA. Genes involved in Aβ metabolism, including A2M, PSEN1, and SORL1, primarily influence AD pathogenesis by regulating Aβ production, transport, and clearance [43]. Moreover, neurotransmitter signaling genes, including BCHE and CHRNA3, whose inhibition is one of the important strategies for combatting AD [43], are critical for acetylcholine metabolism and transport. Furthermore, lipid metabolism‐related genes, including APOC1, play a vital role in the pathological process of AD [44]. Finally, genes associated with Tau protein regulation and apoptosis, such as CASP3, GSK3B, and KCNIP3, contribute to AD pathology. GSK3B may play a role in neuroinflammation [45], neuronal apoptosis, and accumulation of phosphorylated tau in AD [46], while KCNIP3 may play a functional role in the neurobiology of Alzheimer's disease by being part of these gene interaction networks commonly associated with neurodegenerative processes [47].

Finally, receptor mapping revealed significant correlations between PMV and neurotransmitter systems. Our results demonstrated a significant correlation between PMV and 5‐HT receptors, suggesting a potential link to cognitive impairment, as well as declines in learning and memory in AD [48]. Additionally, alterations in CBF were observed, which corresponded to the disease progression of AD. Dopaminergic receptor analysis indicated degradation within the dopamine system, resulting in disrupted dopaminergic signaling, which subsequently impacts cognitive and emotional processes in AD. Furthermore, correlations involving MU, NAT, and SERT revealed alterations in emotional regulation mechanisms. Finally, the observed decline in VAChT, a key factor in acetylcholine storage and release, likely contributes to the memory deficits and cognitive dysfunction characteristic of AD. These processes contribute to the PMV changes observed in AD, further supporting its utility as a biomarker for tracking disease progression.

In conclusion, PMV emerges as a robust, consistent, and sensitive biomarker that captures the nuanced connectivity differences between cortical and subcortical regions. It has the potential to bridge macro‐level connectomics and micro‐level genomics, making it possible to unify external disease manifestations with intrinsic topological changes. However, the PMV is limited by its reliance on a single‐modal network, which restricts its ability to leverage the rich information available from multiple modalities. This may result in the loss of valuable insights that could be captured through a multimodal approach.

## Author Contributions

Juntao Zhao and Yunyun Duan analyzed and performed the measurements; Dawei Wang, Hongxiang Yao, Bo Zhou, Jie Lu, Pan Wang, Zhengluan Liao, Yan Chen, Ying Han, and Xi Zhang collected the data; Juntao Zhao and Kun Zhao were principally responsible for preparing the manuscript; Kun Zhao and Yong Liu revised the manuscript; Kun Zhao, Zhengluan Liao, and Yong Liu supervised the project.

## Conflicts of Interest

The authors declare no conflicts of interest.

## Supporting information


**Figure S1.** Illustration of all 20 motif styles. The edges are colored red to signify strong connections and blue to indicate weak connections. Solid lines represent long‐range connections, while dashed lines denote short‐range connections. Each edge is characterized by properties of connection strength and length, as defined.
**Figure S2.** Scatterplot illustrating the distribution of all 20 motifs across different groups within brain regions. (A) Brain region 84, subregion of the middle temporal gyrus. (B) Brain region 97, subregion of the inferior temporal gyrus. (C) Brain region 103, subregion of the fusiform gyrus. (D) Brain region 117, subregion of the parahippocampal gyrus. (E) Brain region 215, subregion of the hippocampus. (F) Brain region 236, subregion of the thalamus. (*p < 0.05, **p < 0.01, ***p < 0.001).
**Figure S3.** Spatial distributions of the three principal motifs driving PMV variation. (A) Motif 19 (43% variance) shows predominant cortical expression, while (B) Motif 13 (14%) and (C) Motif 14 (12%) demonstrate subcortical localization. Warm colors indicate regions with higher motif representation (color bar at right).
**Figure S4.** Distribution differences with brain functional systems and anatomic regions. Given the non‐normal distributions of all variables, group differences were assessed using the Mann–Whitney U test, with Bonferroni correction applied for multiple comparisons. (ns: not significant (*p* ≥ 0.05), ***p < 0.001).
**Table S1.** The detailed brain regions’ names of the Brainnetome atlas.
**Table S2.** Intensity features describe the distribution of voxel intensities within an MRI image through commonly used and basic metrics.
**Table S3.** Textural features describe the patterns or spatial distribution of voxel intensities.

## Data Availability

All of these datasets were publicly available or had conditional acquisition from the corresponding author. All codes of this study can be found at https://github.com/YongLiuLab.
