# Supplementary material for: Network Topography Alterations in Alzheimer's Disease: Insights From Motif Changes via Multisite Datasets (N = 3262)
Source: CNS Neurosci Ther. 2025 Jun 19;31(6):e70428. doi: 10.1111/cns.70428 (PMC12178941; doi:10.1111/cns.70428)
Supplement: Supplementary file 1 — Figure S1. Illustration of all 20 motif styles. The edges are colored red to signify strong connections and blue to indicate weak connections. Solid lines represent long‐range connections, while dashed lines denote short‐range connections. Each edge is characterized by properties of connection strength and length, as defined. Figure S2. Scatterplot illustrating the distribution of all 20 motifs across different groups within brain regions. (A) Brain region 84, subregion of the middle temporal gyrus. (B) Brain region 97, subregion of the inferior temporal gyrus. (C) Brain region 103, subregion of the fusiform gyrus. (D) Brain region 117, subregion of the parahippocampal gyrus. (E) Brain region 215, subregion of the hippocampus. (F) Brain region 236, subregion of the thalamus. (*p < 0.05, **p < 0.01, ***p < 0.001). Figure S3. Spatial distributions of the three principal motifs driving PMV variation. (A) Motif 19 (43% variance) shows predominant cortical expression, while (B) Motif 13 (14%) and (C) Motif 14 (12%) demonstrate subcortical localization. Warm colors indicate regions with higher motif representation (color bar at right). Figure S4. Distribution differences with brain functional systems and anatomic regions. Given the non‐normal distributions of all variables, group differences were assessed using the Mann–Whitney U test, with Bonferroni correction applied for multiple comparisons. (ns: not significant (p ≥ 0.05), ***p < 0.001). Table S1. The detailed brain regions’ names of the Brainnetome atlas. Table S2. Intensity features describe the distribution of voxel intensities within an MRI image through commonly used and basic metrics. Table S3. Textural features describe the patterns or spatial distribution of voxel intensities. [file CNS-31-e70428-s001.docx]

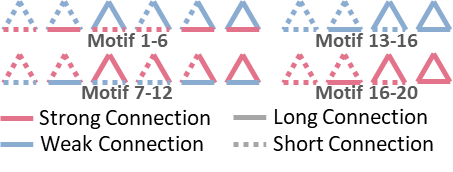


**Figure S01.** Illustration of all 20 motif styles. The edges are colored red to signify strong connections and blue to indicate weak connections. Solid lines represent long-range connections, while dashed lines denote short-range connections. Each edge is characterized by properties of connection strength and length, as defined.


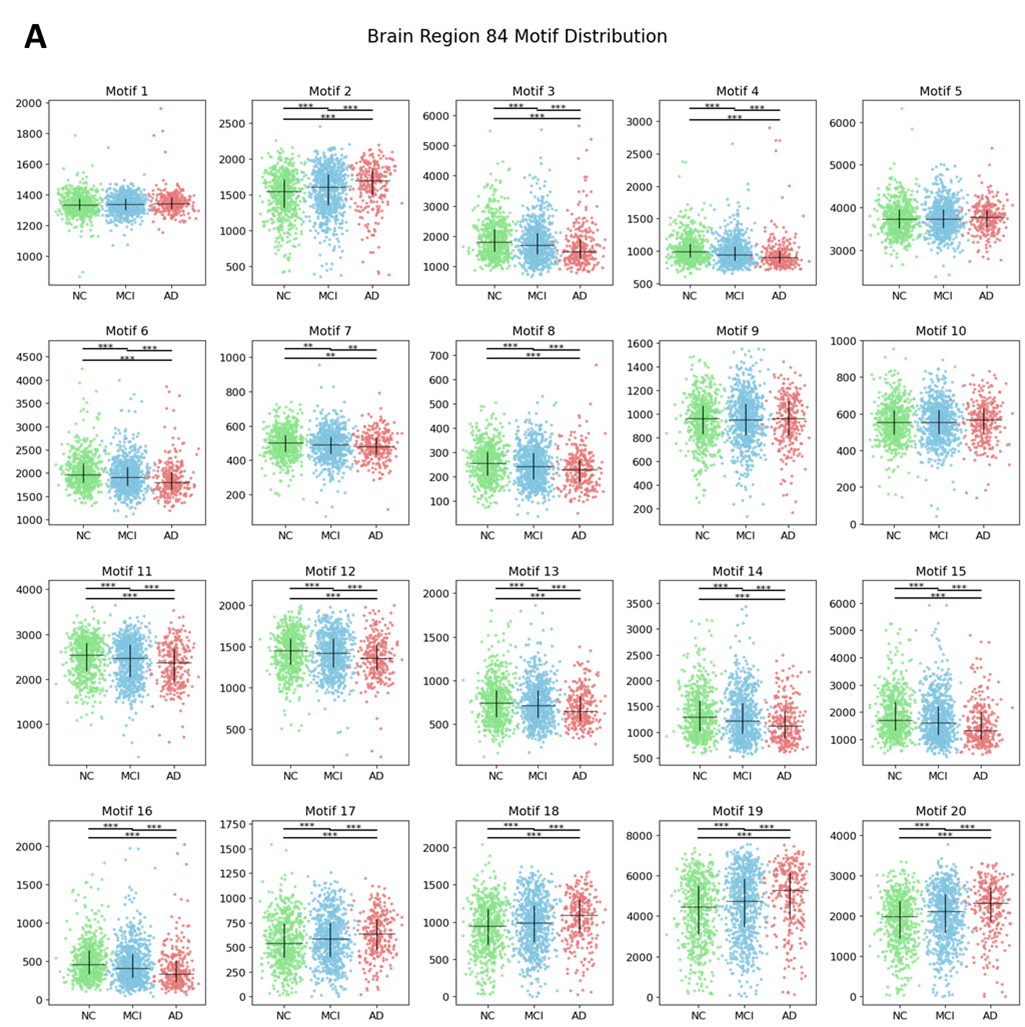


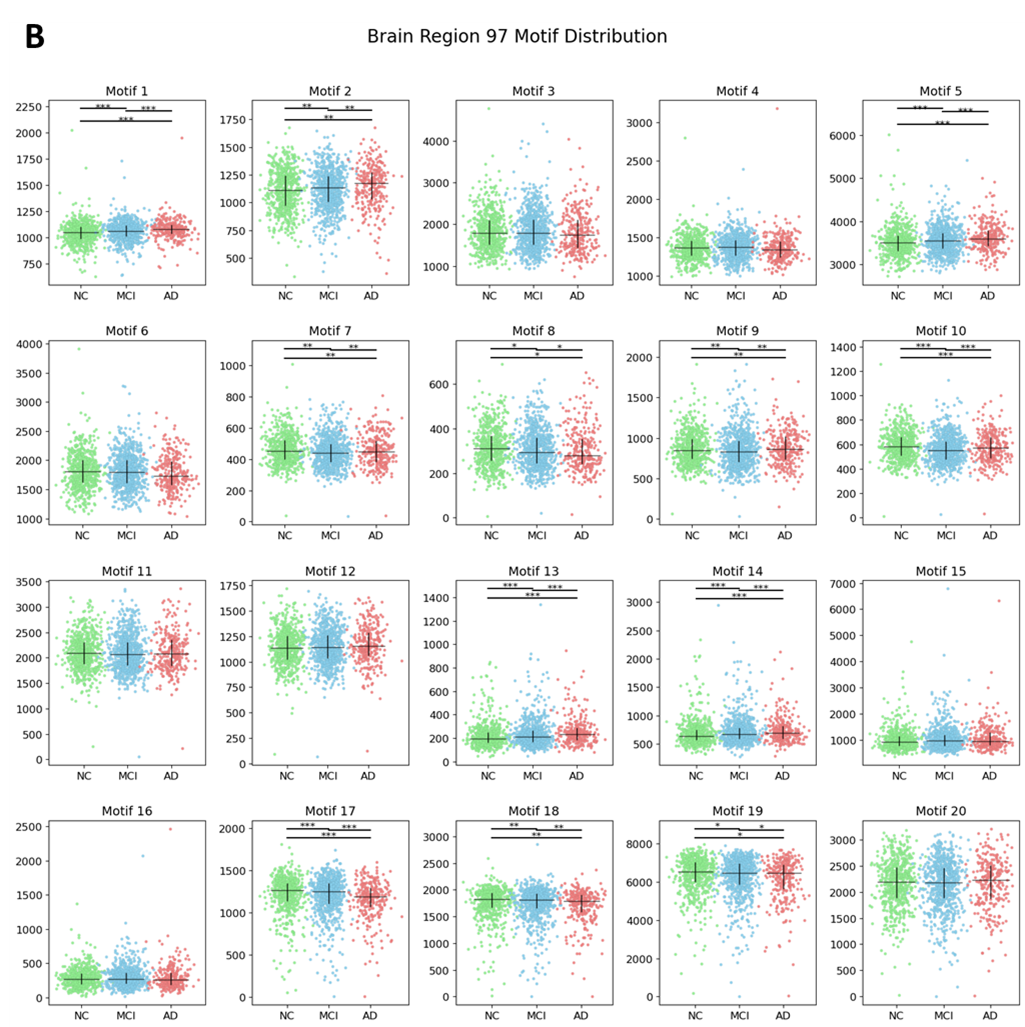

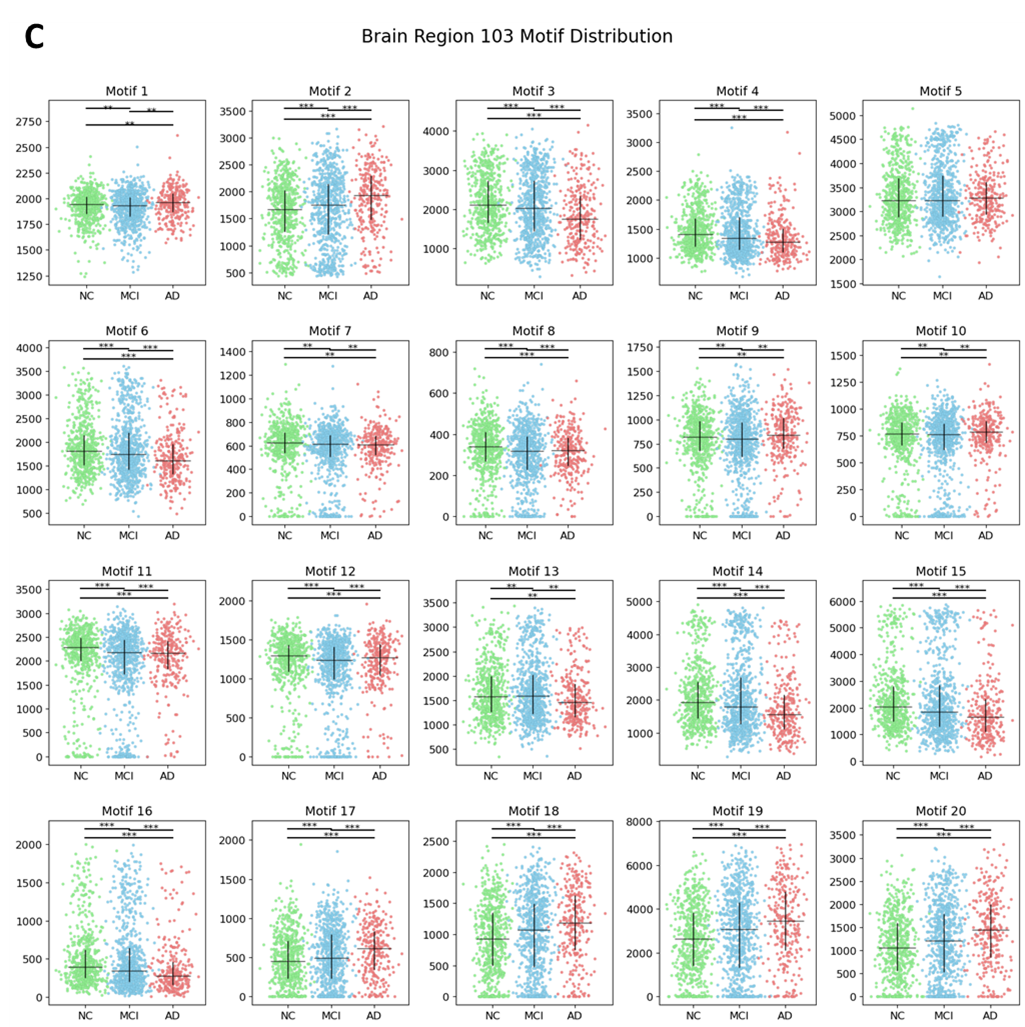

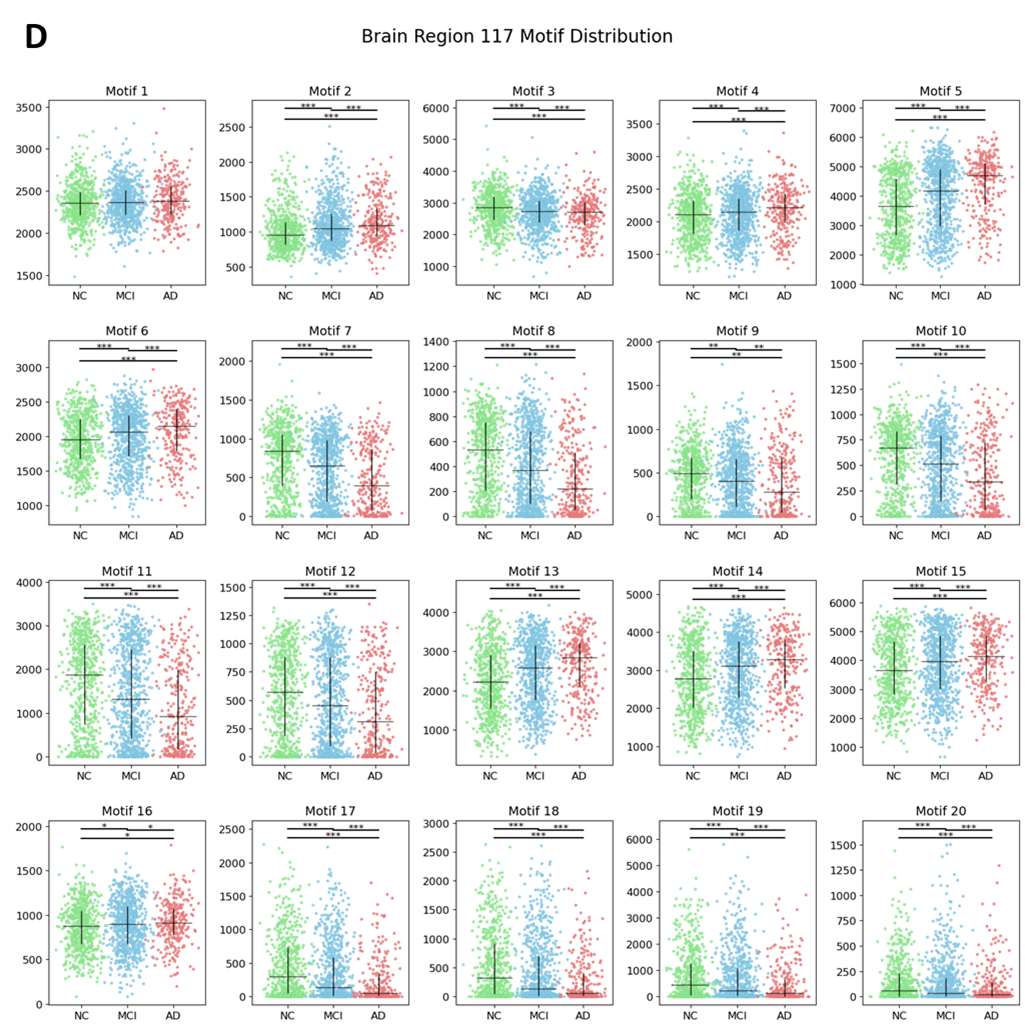

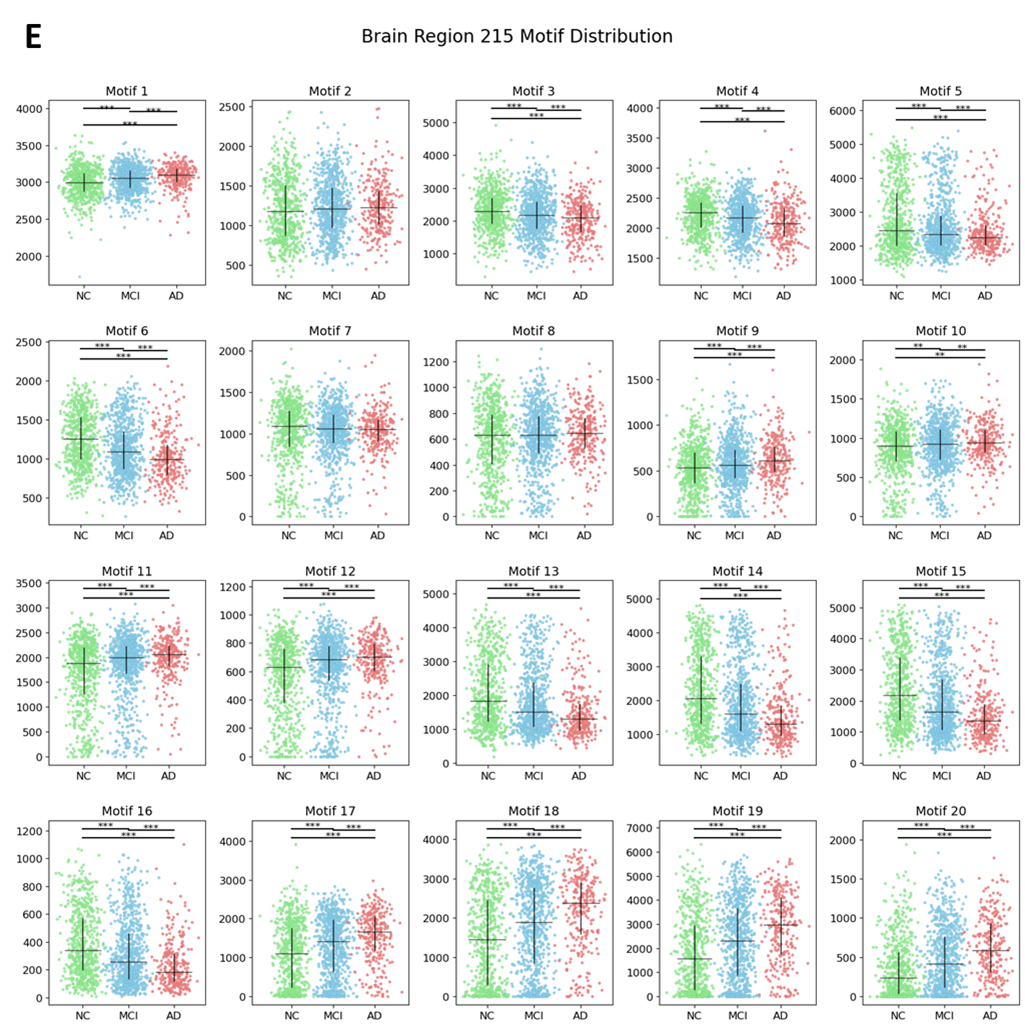

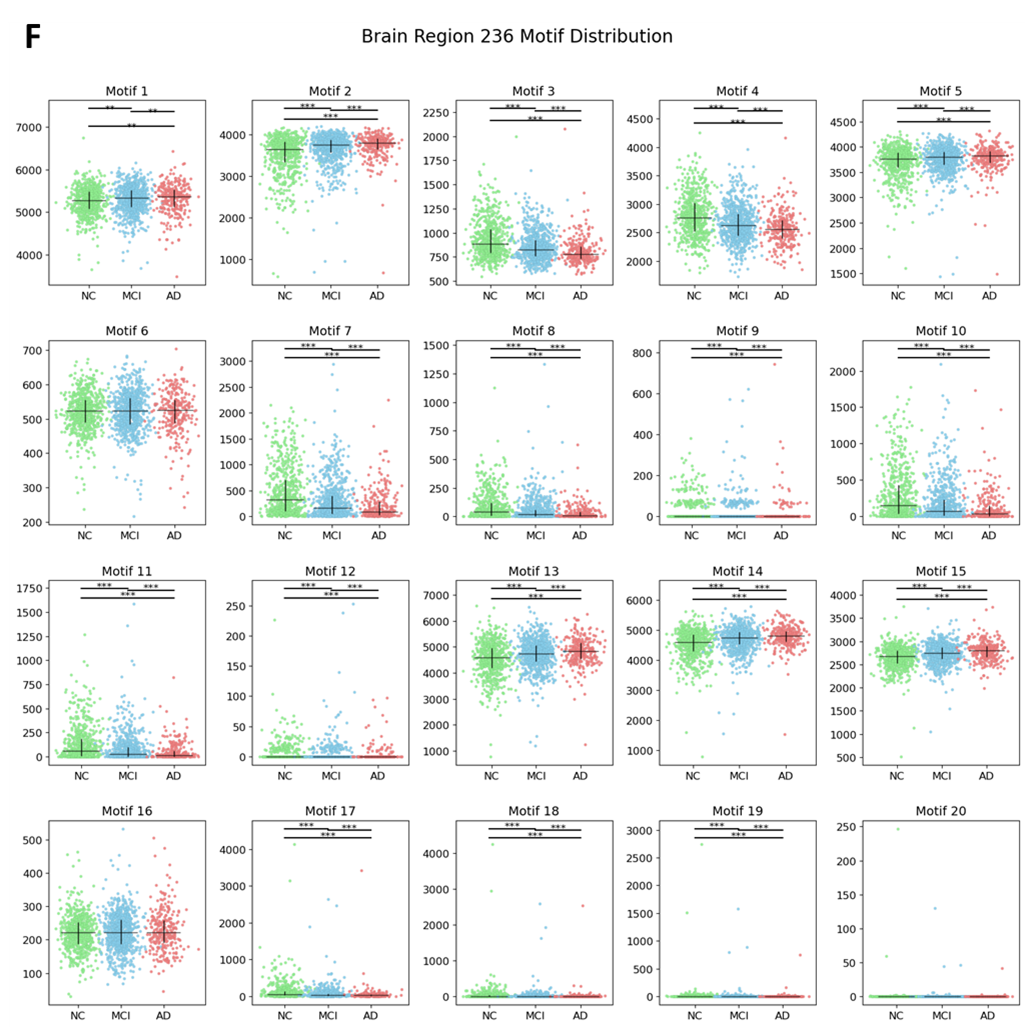


**Figure S02.** Scatterplot illustrating the distribution of all 20 motifs across different groups within brain regions. (A) Brain region 84, subregion of the middle temporal gyrus. (B) Brain region 97, subregion of the inferior temporal gyrus. (C) Brain region 103, subregion of the fusiform gyrus. (D) Brain region 117, subregion of the parahippocampal gyrus. (E) Brain region 215, subregion of the hippocampus. (F) Brain region 236, subregion of the thalamus. (*: p<0.05, **: p<0.01, ***: p < 0.001)


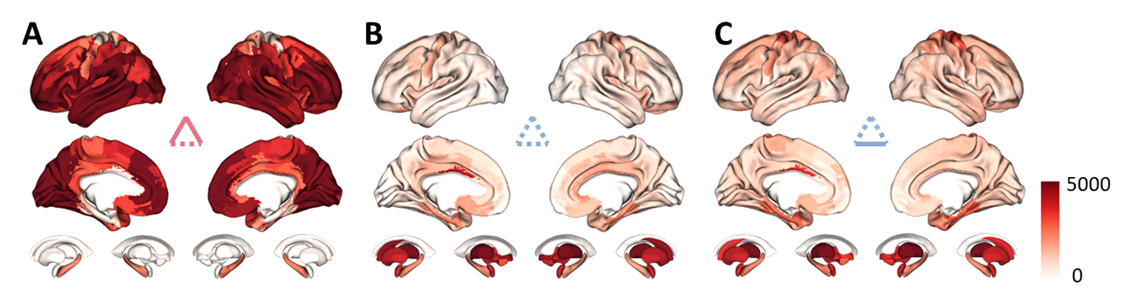


**Fig S03.** Spatial distributions of the three principal motifs driving PMV variation. (A) Motif 19 (43% variance) shows predominant cortical expression, while (B) Motif 13 (14%) and (C) Motif 14 (12%) demonstrate subcortical localization. Warm colors indicate regions with higher motif representation (color bar at right).


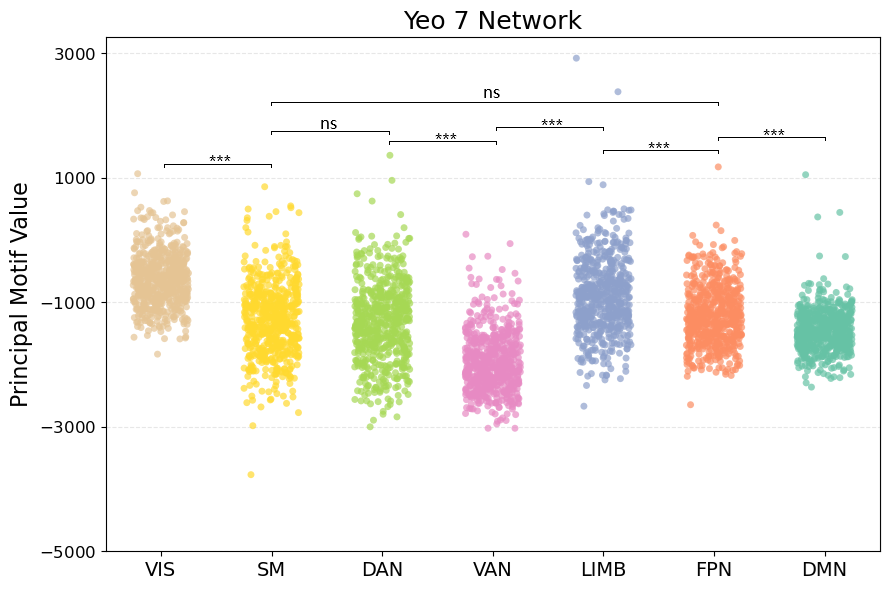

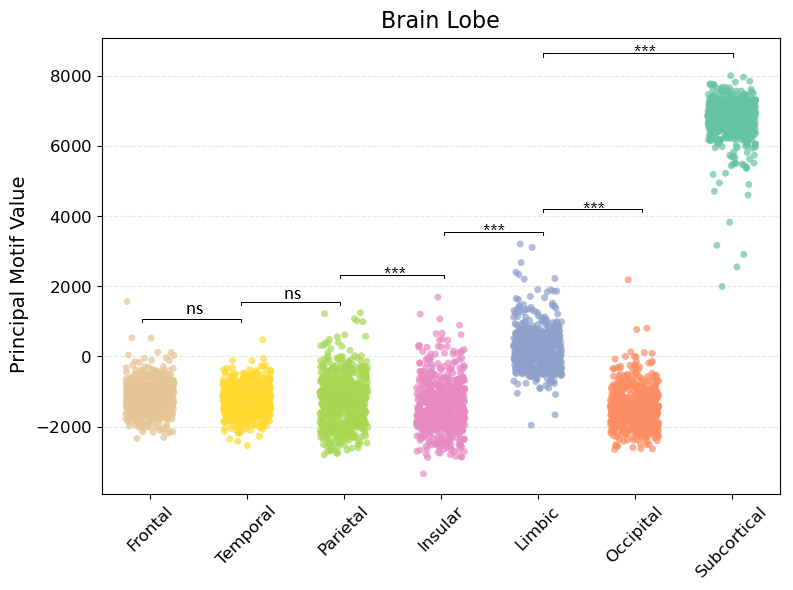


**Fig S04.** Distribution differences with brain functional systems and anatomic regions. Given the non-normal distributions of all variables, group differences were assessed using the Mann-Whitney U test, with Bonferroni correction applied for multiple comparisons. (ns: Not significant (p ≥ 0.05), ***: p < 0.001)

**Table S01.** The detailed brain regions’ names of the Brainnetome atlas.

| **Lobe** | **Gyrus** | **Left and Right Hemisphere** | **Label ID.L** | **Label ID.R** | **Anatomical and modified Cyto-architectonic descriptions** |
| --- | --- | --- | --- | --- | --- |
| **Frontal Lobe** | SFG, Superior Frontal Gyrus | SFG_L(R)_7_1 | 1 | 2 | *A8m, medial area 8* |
|  |  | SFG_L(R)_7_2 | 3 | 4 | *A8dl, dorsolateral area 8* |
|  |  | SFG_L(R)_7_3 | 5 | 6 | *A9l, lateral area 9* |
|  |  | SFG_L(R)_7_4 | 7 | 8 | *A6dl, dorsolateral area 6* |
|  |  | SFG_L(R)_7_5 | 9 | 10 | *A6m, medial area 6* |
|  |  | SFG_L(R)_7_6 | 11 | 12 | *A9m,medial area 9* |
|  |  | SFG_L(R)_7_7 | 13 | 14 | *A10m, medial area 10* |
|  | MFG, Middle Frontal Gyrus | MFG_L(R)_7_1 | 15 | 16 | *A9/46d, dorsal area 9/46* |
|  |  | MFG_L(R)_7_2 | 17 | 18 | *IFJ, inferior frontal junction* |
|  |  | MFG_L(R)_7_3 | 19 | 20 | *A46, area 46* |
|  |  | MFG_L(R)_7_4 | 21 | 22 | *A9/46v, ventral area 9/46* |
|  |  | MFG_L(R)_7_5 | 23 | 24 | *A8vl, ventrolateral area 8* |
|  |  | MFG_L(R)_7_6 | 25 | 26 | *A6vl, ventrolateral area 6* |
|  |  | MFG_L(R)_7_7 | 27 | 28 | *A10l, lateral area10* |
|  | IFG, Inferior Frontal Gyrus | IFG_L(R)_6_1 | 29 | 30 | *A44d,dorsal area 44* |
|  |  | IFG_L(R)_6_2 | 31 | 32 | *IFS, inferior frontal sulcus* |
|  |  | IFG_L(R)_6_3 | 33 | 34 | *A45c, caudal area 45* |
|  |  | IFG_L(R)_6_4 | 35 | 36 | *A45r, rostral area 45* |
|  |  | IFG_L(R)_6_5 | 37 | 38 | *A44op, opercular area 44* |
|  |  | IFG_L(R)_6_6 | 39 | 40 | *A44v, ventral area 44* |
|  | OrG, Orbital Gyrus | OrG_L(R)_6_1 | 41 | 42 | *A14m, medial area 14* |
|  |  | OrG_L(R)_6_2 | 43 | 44 | *A12/47o, orbital area 12/47* |
|  |  | OrG_L(R)_6_3 | 45 | 46 | *A11l, lateral area 11* |
|  |  | OrG_L(R)_6_4 | 47 | 48 | *A11m, medial area 11* |
|  |  | OrG_L(R)_6_5 | 49 | 50 | *A13, area 13* |
|  |  | OrG_L(R)_6_6 | 51 | 52 | *A12/47l, lateral area 12/47* |
|  | PrG, Precentral Gyrus | PrG_L(R)_6_1 | 53 | 54 | *A4hf, area 4(head and face region)* |
|  |  | PrG_L(R)_6_2 | 55 | 56 | *A6cdl, caudal dorsolateral area 6* |
|  |  | PrG_L(R)_6_3 | 57 | 58 | *A4ul, area 4(upper limb region)* |
|  |  | PrG_L(R)_6_4 | 59 | 60 | *A4t, area 4(trunk region)* |
|  |  | PrG_L(R)_6_5 | 61 | 62 | *A4tl, area 4(tongue and larynx region)* |
|  |  | PrG_L(R)_6_6 | 63 | 64 | *A6cvl, caudal ventrolateral area 6* |
|  | PCL, Paracentral Lobule | PCL_L(R)_2_1 | 65 | 66 | *A1/2/3ll, area1/2/3 (lower limb region)* |
|  |  | PCL_L(R)_2_2 | 67 | 68 | *A4ll, area 4, (lower limb region)* |
| **Temporal Lobe** | STG, Superior Temporal Gyrus | STG_L(R)_6_1 | 69 | 70 | *A38m, medial area 38* |
|  |  | STG_L(R)_6_2 | 71 | 72 | *A41/42, area 41/42* |
|  |  | STG_L(R)_6_3 | 73 | 74 | *TE1.0 and TE1.2* |
|  |  | STG_L(R)_6_4 | 75 | 76 | *A22c, caudal area 22* |
|  |  | STG_L(R)_6_5 | 77 | 78 | *A38l, lateral area 38* |
|  |  | STG_L(R)_6_6 | 79 | 80 | *A22r, rostral area 22* |
|  | MTG, Middle Temporal Gyrus | MTG_L(R)_4_1 | 81 | 82 | *A21c, caudal area 21* |
|  |  | MTG_L(R)_4_2 | 83 | 84 | *A21r, rostral area 21* |
|  |  | MTG_L(R)_4_3 | 85 | 86 | *A37dl, dorsolateral area37* |
|  |  | MTG_L(R)_4_4 | 87 | 88 | *aSTS, anterior superior temporal sulcus* |
|  | ITG, Inferior Temporal Gyrus | ITG_L(R)_7_1 | 89 | 90 | *A20iv, intermediate ventral area 20* |
|  |  | ITG_L(R)_7_2 | 91 | 92 | *A37elv, extreme lateroventral area37* |
|  |  | ITG_L(R)_7_3 | 93 | 94 | *A20r, rostral area 20* |
|  |  | ITG_L(R)_7_4 | 95 | 96 | *A20il, intermediate lateral area 20* |
|  |  | ITG_L(R)_7_5 | 97 | 98 | *A37vl, ventrolateral area 37* |
|  |  | ITG_L(R)_7_6 | 99 | 100 | *A20cl, caudolateral of area 20* |
|  |  | ITG_L(R)_7_7 | 101 | 102 | *A20cv, caudoventral of area 20* |
|  | FuG, Fusiform Gyrus | FuG_L(R)_3_1 | 103 | 104 | *A20rv, rostroventral area 20* |
|  |  | FuG_L(R)_3_2 | 105 | 106 | *A37mv, medioventral area37* |
|  |  | FuG_L(R)_3_3 | 107 | 108 | *A37lv, lateroventral area37* |
|  | PhG, Parahippocampal Gyrus | PhG_L(R)_6_1 | 109 | 110 | *A35/36r, rostral area 35/36* |
|  |  | PhG_L(R)_6_2 | 111 | 112 | *A35/36c, caudal area 35/36* |
|  |  | PhG_L(R)_6_3 | 113 | 114 | *TL, area TL (lateral PPHC, posterior parahippocampal gyrus)* |
|  |  | PhG_L(R)_6_4 | 115 | 116 | *A28/34, area 28/34 (EC, entorhinal cortex)* |
|  |  | PhG_L(R)_6_5 | 117 | 118 | *TI, area TI(temporal agranular insular cortex)* |
|  |  | PhG_L(R)_6_6 | 119 | 120 | *TH, area TH (medial PPHC)* |
|  | pSTS, posterior Superior Temporal Sulcus | pSTS_L(R)_2_1 | 121 | 122 | *rpSTS, rostroposterior superior temporal sulcus* |
|  |  | pSTS_L(R)_2_2 | 123 | 124 | *cpSTS, caudoposterior superior temporal sulcus* |
| **Parietal Lobe** | SPL, Superior Parietal Lobule | SPL_L(R)_5_1 | 125 | 126 | *A7r, rostral area 7* |
|  |  | SPL_L(R)_5_2 | 127 | 128 | *A7c, caudal area 7* |
|  |  | SPL_L(R)_5_3 | 129 | 130 | *A5l, lateral area 5* |
|  |  | SPL_L(R)_5_4 | 131 | 132 | *A7pc, postcentral area 7* |
|  |  | SPL_L(R)_5_5 | 133 | 134 | *A7ip, intraparietal area 7(hIP3)* |
|  | IPL, Inferior Parietal Lobule | IPL_L(R)_6_1 | 135 | 136 | *A39c, caudal area 39(PGp)* |
|  |  | IPL_L(R)_6_2 | 137 | 138 | *A39rd, rostrodorsal area 39(Hip3)* |
|  |  | IPL_L(R)_6_3 | 139 | 140 | *A40rd, rostrodorsal area 40(PFt)* |
|  |  | IPL_L(R)_6_4 | 141 | 142 | *A40c, caudal area 40(PFm)* |
|  |  | IPL_L(R)_6_5 | 143 | 144 | *A39rv, rostroventral area 39(PGa)* |
|  |  | IPL_L(R)_6_6 | 145 | 146 | *A40rv, rostroventral area 40(PFop)* |
|  | Pcun, Precuneus | PCun_L(R)_4_1 | 147 | 148 | *A7m, medial area 7(PEp)* |
|  |  | PCun_L(R)_4_2 | 149 | 150 | *A5m, medial area 5(PEm)* |
|  |  | PCun_L(R)_4_3 | 151 | 152 | *dmPOS, dorsomedial parietooccipital sulcus(PEr)* |
|  |  | PCun_L(R)_4_4 | 153 | 154 | *A31, area 31 (Lc1)* |
|  | PoG, Postcentral Gyrus | PoG_L(R)_4_1 | 155 | 156 | *A1/2/3ulhf, area 1/2/3(upper limb, head and face region)* |
|  |  | PoG_L(R)_4_2 | 157 | 158 | *A1/2/3tonIa, area 1/2/3(tongue and larynx region)* |
|  |  | PoG_L(R)_4_3 | 159 | 160 | *A2, area 2* |
|  |  | PoG_L(R)_4_4 | 161 | 162 | *A1/2/3tru, area1/2/3(trunk region)* |
| **Insular Lobe** | INS, Insular Gyrus | INS_L(R)_6_1 | 163 | 164 | *G, hypergranular insula* |
|  |  | INS_L(R)_6_2 | 165 | 166 | *vIa, ventral agranular insula* |
|  |  | INS_L(R)_6_3 | 167 | 168 | *dIa, dorsal agranular insula* |
|  |  | INS_L(R)_6_4 | 169 | 170 | *vId/vIg, ventral dysgranular and granular insula* |
|  |  | INS_L(R)_6_5 | 171 | 172 | *dIg, dorsal granular insula* |
|  |  | INS_L(R)_6_6 | 173 | 174 | *dId, dorsal dysgranular insula* |
| **Limbic Lobe** | CG, Cingulate Gyrus | CG_L(R)_7_1 | 175 | 176 | *A23d, dorsal area 23* |
|  |  | CG_L(R)_7_2 | 177 | 178 | *A24rv, rostroventral area 24* |
|  |  | CG_L(R)_7_3 | 179 | 180 | *A32p, pregenual area 32* |
|  |  | CG_L(R)_7_4 | 181 | 182 | *A23v, ventral area 23* |
|  |  | CG_L(R)_7_5 | 183 | 184 | *A24cd, caudodorsal area 24* |
|  |  | CG_L(R)_7_6 | 185 | 186 | *A23c, caudal area 23* |
|  |  | CG_L(R)_7_7 | 187 | 188 | *A32sg, subgenual area 32* |
| **Occipital Lobe** | MVOcC*,* MedioVentral Occipital Cortex | MVOcC _L(R)_5_1 | 189 | 190 | *cLinG, caudal lingual gyrus* |
|  |  | MVOcC _L(R)_5_2 | 191 | 192 | *rCunG, rostral cuneus gyrus* |
|  |  | MVOcC _L(R)_5_3 | 193 | 194 | *cCunG, caudal cuneus gyrus* |
|  |  | MVOcC _L(R)_5_4 | 195 | 196 | *rLinG, rostral lingual gyrus* |
|  |  | MVOcC _L(R)_5_5 | 197 | 198 | *vmPOS,ventromedial parietooccipital sulcus* |
|  | LOcC, lateral Occipital Cortex | LOcC_L(R)_4_1 | 199 | 200 | *mOccG, middle occipital gyrus* |
|  |  | LOcC _L(R)_4_2 | 201 | 202 | *V5/MT+, area V5/MT+* |
|  |  | LOcC _L(R)_4_3 | 203 | 204 | *OPC, occipital polar cortex* |
|  |  | LOcC_L(R)_4_4 | 205 | 206 | *iOccG, inferior occipital gyrus* |
|  |  | LOcC _L(R)_2_1 | 207 | 208 | *msOccG, medial superior occipital gyrus* |
|  |  | LOcC _L(R)_2_2 | 209 | 210 | *lsOccG, lateral superior occipital gyrus* |
| **Subcortical Nuclei** | Amyg, Amygdala | Amyg_L(R)_2_1 | 211 | 212 | *mAmyg, medial amygdala* |
|  |  | Amyg_L(R)_2_2 | 213 | 214 | *lAmyg, lateral amygdala* |
|  | Hipp, Hippocampus | Hipp_L(R)_2_1 | 215 | 216 | *rHipp, rostral hippocampus* |
|  |  | Hipp_L(R)_2_2 | 217 | 218 | *cHipp, caudal hippocampus* |
|  | BG, Basal Ganglia | BG_L(R)_6_1 | 219 | 220 | *vCa, ventral caudate* |
|  |  | BG_L(R)_6_2 | 221 | 222 | *GP, globus pallidus* |
|  |  | BG_L(R)_6_3 | 223 | 224 | *NAC, nucleus accumbens* |
|  |  | BG_L(R)_6_4 | 225 | 226 | *vmPu, ventromedial putamen* |
|  |  | BG_L(R)_6_5 | 227 | 228 | *dCa, dorsal caudate* |
|  |  | BG_L(R)_6_6 | 229 | 230 | *dlPu, dorsolateral putamen* |
|  | Tha, Thalamus | Tha_L(R)_8_1 | 231 | 232 | *mPFtha, medial pre-frontal thalamus* |
|  |  | Tha_L(R)_8_2 | 233 | 234 | *mPMtha, pre-motor thalamus* |
|  |  | Tha_L(R)_8_3 | 235 | 236 | *Stha, sensory thalamus* |
|  |  | Tha_L(R)_8_4 | 237 | 238 | *rTtha, rostral temporal thalamus* |
|  |  | Tha_L(R)_8_5 | 239 | 240 | *PPtha, posterior parietal thalamus* |
|  |  | Tha_L(R)_8_6 | 241 | 242 | *Otha, occipital thalamus* |
|  |  | Tha_L(R)_8_7 | 243 | 244 | *cTtha, caudal temporal thalamus* |
|  |  | Tha_L(R)_8_8 | 245 | 246 | *lPFtha, lateral pre-frontal thalamus* |

**Table S02.** Intensity features describe the distribution of voxel intensities within an MRI image through commonly used and basic metrics.

|  | Image feature | Equation | Definition |
| --- | --- | --- | --- |
| Intensity  Features  (14) | Energy | $\sum_{i}^{N} X(i)^{2}$ | Measure of the randomness of the intensity values in an image |
|  | Entropy | $\sum_{i=1}^{N_{l}} P(i)\mathrm{lo}g_{2}P(i)$ | Represents the irregularity of the intensity value distribution |
|  | Kurtosis | $\frac{\frac{1}{N}\Sigma_{i=1}^{N}(X(i)-\bar{X})^{4}}{\left( \sqrt{\frac{1}{N}\Sigma_{i=1}^{N}(X(i)-\bar{X})^{2}} \right)^{2}}$ | The peakedness of the histogram or indication of histogram flatness |
|  | Maximum | Maximum intensity value of X |  |
|  | Mean | $\frac{1}{N}\sum_{i}^{N} X(i)$ | Average intensity value of the pixels within the region ofinterest |
|  | Mean Absolute Deviation(mad) | Mean of the absolute deviations of all voxel intensities around the mean intensity value | A measure of how much the gray levels differ from the mean |
|  | Median | Median intensity value of X |  |
|  | Minimum | Minimum intensity value of X |  |
|  | Range | Range of intensity values of X |  |
|  | Root Mean Square(RMS) | $\sqrt{\frac{\sum_{i}^{N} X(i)^{2}}{N}}$ |  |
|  | Skewness | $\frac{\frac{1}{N}\sum_{i=1}^{N} (X(i)-\bar{X})^{3}}{\left( \sqrt{\frac{1}{N}\sum_{i=1}^{N} (X(i)-\bar{X})^{2}} \right)^{3}}$ | Symmetry of intensity values in an image |
|  | Standard Deviation | $\left( \frac{1}{N-1}\sum_{i=1}^{N} (X(i)-\bar{X})^{2} \right)^{1/2}$ | A measure of how much variation or dispersion exists |
|  | Uniformity | $\sum_{i=1}^{N_{l}} P(i)^{2}$ | Measures the homogeneity of the intensity value distribution in an image |
|  | Variance(Var) | $\frac{1}{N-1}\sum_{i=1}^{N} (X(i)-\bar{X})^{2}$ | The spread or variation around the mean (sum of squares) |

$X$ denotes the three-dimensional image matrix. $N$ is the number of voxels. $P$ is the first-order histogram with $N_{l}$ discrete intensity levels. $\bar{X}$ is the mean of $X$. The number of histogram bins is 100.

**Table S03.** Textural features describe the patterns or spatial distribution of voxel intensities.

|  | Image feature | Equation | Definition |
| --- | --- | --- | --- |
| Textural  features  (33) | Autocorrelation | $\sum_{i=1}^{N_{g}} \sum_{j=1}^{N_{g}} ijP(i,j)$ |  |
|  | Cluster Prominence (CP) | $\sum_{i=1}^{N_{g}} \sum_{j=1}^{N_{g}} [i+j-\mu_{x}(i)-\mu_{y}(j)]^{4}P(i,j)$ |  |
|  | Cluster Shade | $\sum_{i=1}^{N_{g}} \sum_{j=1}^{N_{g}} [i+j-\mu_{x}(i)-\mu_{y}(j)]^{3}P(i,j)$ |  |
|  | Cluster Tendency | $\sum_{i=1}^{N_{g}} \sum_{j=1}^{N_{g}} [i+j-\mu_{x}(i)-\mu_{y}(j)]^{2}P(i,j)$ |  |
|  | Contrast | $\sum_{i=1}^{N_{g}} \sum_{j=1}^{N_{g}} \vert i-j\vert^{2}P(i,j)$ | Measures the local variation inintensity values |
|  | Correlation | $\frac{\sum_{i=1}^{N_{g}} \sum_{j=1}^{N_{g}} ijP(i,j)-\mu_{i}(i)\mu_{j}(j)}{\sigma_{x}(i)\sigma_{y}(j)}$ | Measures the linear dependencies of intensity values in an image |
|  | Difference Entropy | $\sum_{i=0}^{N_{g}-1} P_{x-y}(i)log_{2}[P_{x-y}(i)]$ |  |
|  | Dissimilarity | $\sum_{i=1}^{N_{g}} \sum_{j=1}^{N_{g}} \vert i-j\vert P(i,j)$ |  |
|  | Energy | $\sum_{i=1}^{N_{g}} \sum_{j=1}^{N_{g}} [P(i,j)]^{2}$ |  |
|  | Entropy | $-\sum_{i=1}^{N_{g}} \sum_{j=1}^{N_{g}} P(i,j)\log_{2}[P(i,j)]$ |  |
|  | Homogeneity1 | $\sum_{i=1}^{N_{g}} \sum_{j=1}^{N_{g}} \frac{P(i,j)}{1+\vert i-j\vert}$ | Measures the homogeneity of the intensity values |
|  | Homogeneity2 | $\sum_{i=1}^{N_{g}} \sum_{j=1}^{N_{g}} \frac{P(i,j)}{1+\vert i-j\vert^{2}}$ | Measures the homogeneity of the intensity values of the pixel pair |
|  | Informational Measure of Correlation 1 (IMC1) | $\frac{HXY-HXY1}{max\{HX,HY\}}$ |  |
|  | Informational Measure of Correlation 2 (IMC2) | $\sqrt{1-e^{-2(HXY2-HXY)}}$ |  |
|  | Inverse Difference Moment Normalized (IDMN) | $\sum_{i=1}^{N_{g}} \sum_{j=1}^{N_{g}} \frac{P(i,j)}{1+\left( \frac{\vert i-j\vert^{2}}{N^{2}} \right)}$ |  |
|  | Inverse Difference Normalized (IDN) | $\sum_{i=1}^{N_{g}} \sum_{j=1}^{N_{g}} \frac{P(i,j)}{1+\left( \frac{\vert i-j\vert}{N} \right)}$ |  |
|  | Inverse Variance | $\sum_{i=1}^{N_{g}} \sum_{j=1}^{N_{g}} \frac{P(i,j)}{\vert i-j\vert^{2}},i\neq j$ |  |
|  | Maximum Probability | $max\{P(i,j)\}$ |  |
|  | Sum Average | $\sum_{i=2}^{2N_{g}} [iP_{x+y}(i)]$ |  |
|  | Sum Entropy | $-\sum_{i=2}^{2N_{g}} P_{x+y}(i)log_{2}[P_{x+y}(i)]$ |  |
|  | Sum Variance | $\sum_{i=2}^{2N_{g}} (i-SE)^{2}P_{x+y}(i)$ |  |
|  | Variance | $\sum_{i=1}^{N_{g}} \sum_{j=1}^{N_{g}} (i-\mu)^{2}P(i,j)$ |  |
|  | Short Run Emphasis (SRE) | $\frac{\sum_{i=1}^{N_{g}} \sum_{j=1}^{N_{r}} \left[ \frac{p(i,j\vert\theta)}{j^{2}} \right]}{\sum_{i=1}^{N_{g}} \sum_{j=1}^{N_{r}} p(i,j\vert\theta)}$ |  |
|  | Long Run Emphasis (LRE) | $\frac{\sum_{i=1}^{N_{g}} \sum_{j=1}^{N_{r}} j^{2}p(i,j\vert\theta)}{\sum_{i=1}^{N_{g}} \sum_{j=1}^{N_{r}} p(i,j\vert\theta)}$ |  |
|  | Gray Level Nonuniformity (GLN) | $\frac{\sum_{i=1}^{N_{g}} \left[ \sum_{j=1}^{N_{r}} p(i,j\vert\theta) \right]^{2}}{\sum_{i=1}^{N_{g}} \sum_{j=1}^{N_{r}} p(i,j\vert\theta)}$ | Represents the similarity of intensity values in an image |

$P(i,j)$ is the co-occurrence matrix for an arbitrary δ and α

$p(i,j|\theta)$ is the $(i,j)$th entry in the given run-length matrix $p$ for a direction $\theta$

$N_{g}$ is the number of discrete intensity values in the image

$N_{r}$ is the number of different run lengths

$N_{p}$ is the number of voxels in the image

$\mu$ is the mean of $P(i,j)$

$P_{x}\left( i \right)=\sum_{j=1}^{N_{g}} P(i,j)$ is the marginal row probabilities

$P_{y}\left( i \right)=\sum_{i=1}^{N_{g}} P(i,j)$ is the marginal column probabilities

$\mu_{x}$ is the mean of $p_{x}$

$\mu_{y}$ is the mean of $p_{y}$

$\sigma_{x}$ is the standard deviation of $p_{x}$

$\sigma_{y}$ is the standard deviation of $p_{y}$

$P_{x+y}\left( k \right)=\sum_{i=1}^{N_{g}} \sum_{j=1}^{N_{g}} P(i,j)$, i+j=k, k=2,3,…,${2\times N}_{g}$

$P_{x-y}\left( k \right)=\sum_{i=1}^{N_{g}} \sum_{j=1}^{N_{g}} P(i,j)$, |i-j|=k, k=0,1,…,$N_{g}-1$

HX=$-\sum_{i=1}^{N_{g}} p_{x}(i)\mathrm{lo}g_{2}[p_{x}(i)]$ is the entropy of $p_{x}$

HY=$-\sum_{i=1}^{N_{g}} p_{y}(i)\mathrm{lo}g_{2}[p_{y}(i)]$ is the entropy of $p_{y}$

H=$-\sum_{i=1}^{N_{g}} \sum_{j=1}^{N_{g}} P(i,j)\log_{2}[P(i,j)]$ is the entropy of $P(i,j)$

HXY1=$-\sum_{i=1}^{N_{g}} \sum_{j=1}^{N_{g}} P(i,j)log\left( p_{x}(i)p_{y}(j) \right)$

HXY2=$-\sum_{i=1}^{N_{g}} \sum_{j=1}^{N_{g}} p_{x}(i)p_{y}(j)log\left( p_{x}(i)p_{y}(j) \right)$
